# Total onychodystrophy secondary to complex regional pain syndrome type II: a rare neurotrophic nail disorder^؆^

**DOI:** 10.1016/j.abd.2026.501391

**Published:** 2026-06-18

**Authors:** Elisandra Bárbara Pontes Carlos, Ivanka Miranda de Castro Martins, Fábio Antônio de Andrade, Laís Guimarães Souza, Anna Carolina Miola

**Affiliations:** Department of Infectology, Dermatology, Imaging Diagnosis and Radiotherapy, Faculty of Medicine, Universidade Estadual Paulista, Botucatu, SP, Brazil

Dear Editor,

Complex Regional Pain Syndrome (CRPS), previously named as Reflex Sympathetic Dystrophy (RSD), is a neurological disorder characterized by chronic and continuous pain, added to other neurovascular signs, typically affecting the extremities, caused by noxious events or neural injury.^1^ CRPS is characterized by pain, edema, vasomotor instability, skin changes, and irregular bone demineralization. Skin changes related to CRPS are common and result from dysregulated sympathetic activity and microvascular changes.^2^ Nails are also susceptible to developing abnormalities in this context.

A 20-year-old man, three months after a motorcycle accident which resulted in complete brachial plexus injury with avulsion of C5, C6, C7, and C8 roots, developed nail changes spontaneously in his right hand, followed by episodic pain crises and spontaneous ulcers in the ipsilateral arm (Fig. 1, Fig. 2). The ulcers exhibited hyperpigmented borders, absence of exudation, and clean bases, without fibrin deposition or other significant clinical signs of infection (Fig. 3). Dermatological examination revealed total onychodystrophy affecting all five fingernails of the affected hand (Fig. 4). The contralateral hand was not affected.

**Fig. 1:**
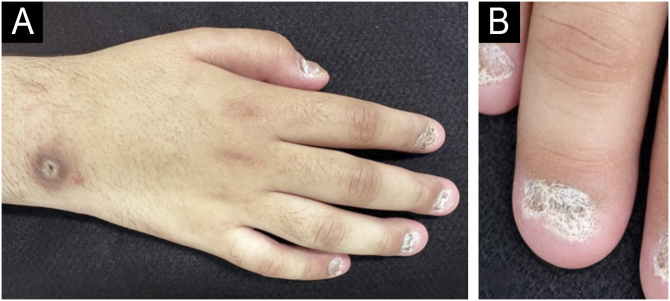
(A) Spontaneous ulcer on the right arm, with total onychodystrophy of fingernails. (B) Magnification of nail onychodistrophy of the fourth finger, showing complete dystrophy of the nail plate.

**Fig. 2:**
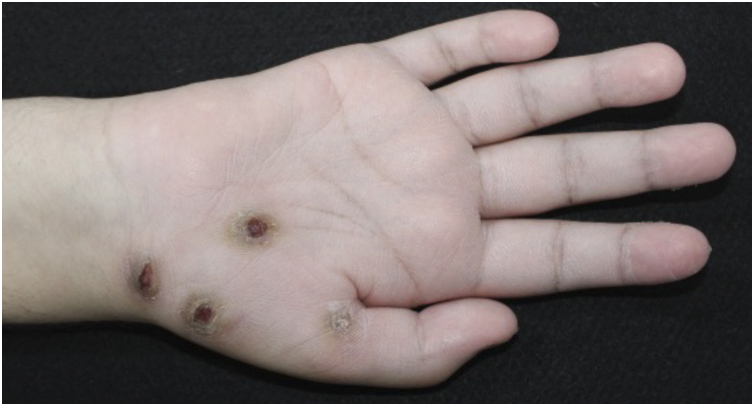
Presence of four rounded ulcers with hyperkeratosis at the edges and a hemorrhagic crust in the center. Note the desquamation on the second finger, likely due to local dysautonomia.

**Fig. 3:**
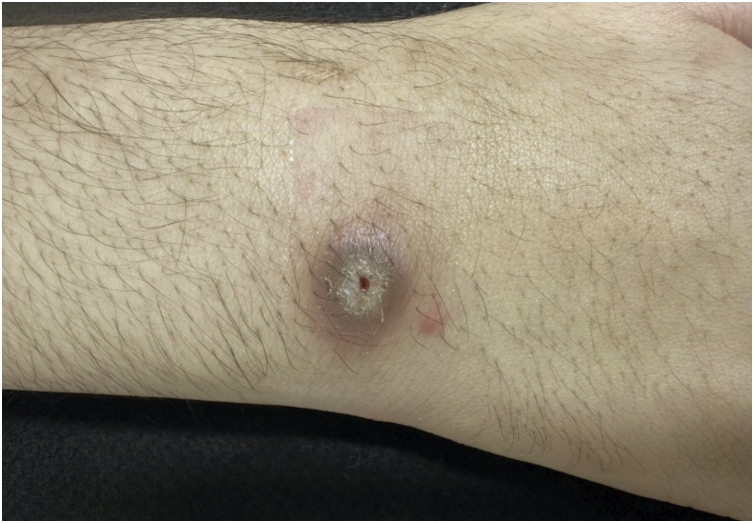
Zoom of the ulcer, revealing more details: hyperchromic borders and absense of exsudation. The base of the punctiform ulcer is clean.

**Fig. 4:**
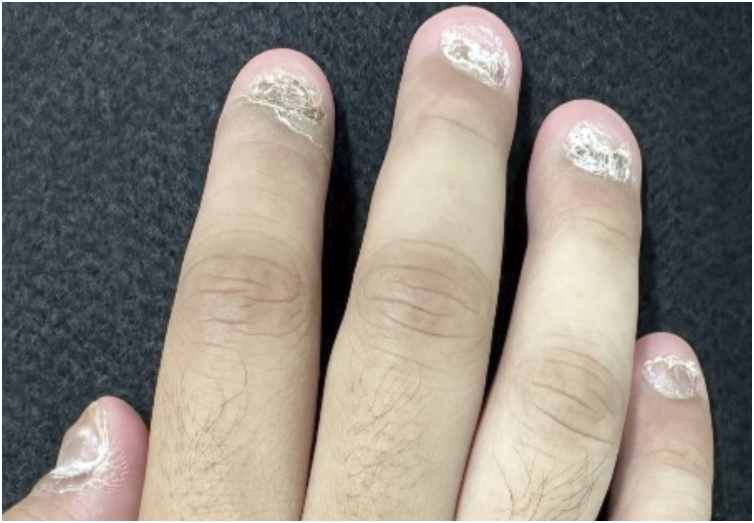
Total onychodystrophy of all five fingernails on the right hand, evidencing the absence of the nail plate in some digits.

Direct mycological examination and fungal culture of the nails and skin lesions were negative. Histopathological examination of an ulcerated lesion demonstrated reactive epidermal hyperplasia, underlying granulation tissue, and a mild perivascular lymphocytic infiltrate, with no evidence of fungal elements on Gomori-Grocott methenamine silver staining. Histopathological exam of the nail unit was not performed, considering that the onychodystrophy was limited to a single hand, rendering alternative diagnoses such as psoriasis or nail lichen planus unlikely.^3^

Considering the unilateral distribution, temporal relationship with the neural injury, absence of structures and associated trophic and vasomotor alterations, the diagnosis of total onychodystrophy secondary to CRPS type II was established, and the hypothesis of dystrophic onychomycosis and sporothrichosis due to the ascending lesions was excluded.

CRPS is a neurological disorder divided into two types: type I, which follows a noxious event without an identifiable nerve lesion; and type II, caused by nerve injury, such as trauma or surgery.^1^ According to the Budapest criteria, diagnosis requires pain disproportionate to the inciting event, with at least one symptom in three of four categories (sensory, vasomotor, sudomotor/edema, and motor/trophic) and one sign in two or more categories.^4^ In our case, the patient presented sensory abnormalities (pain), vasomotor and sudomotor disturbances (episodic edema and ulcers), and trophic changes (onychodystrophy). Skin changes may be classified as vasomotor changes, when involving alterations in skin color, or motor/trophic changes in the skin and nails.^2^

Regarding nail alterations, leukonychia, Beau's lines, nail-fold swelling, clubbing, acute paronychia, dystrophy and trachonychia secondary to CRPS were already related.^5^, ^6^, ^7^ Onychodystrophy has only been reported once before in the literature, of lesser intensity.^8^ It is essential to exclude onychomycosis, the main differential diagnosis, using direct mycological examination and culture, which were negative in our case.

Previous reports have suggested that sympathetic denervation and matrix edema underlie Beau’s lines and trachonychia in CRPS,^5^ but there are no studies in the literature describing the etiopathology of the total onychodystrophy resulting from CRPS. Neurological dysregulation and altered microvascular changes may be involved in the development of these alterations, given that the nail matrix is highly vascularized, and its blood supply can be impaired in patients with vascular compromise secondary to neural lesions, such as in CRPS.^9^

Besides, there is a correlation between embryologic development of nail apparatus and nervous system, both originating from the ectoderm, which may have complex and intimate interactions not yet studied between the structures.^10^ Trachonychia, a matrix nail disorder, in CRPS is due to disrupted nail matrix differentiation and maturation, resulting in the disordered arrangement of keratinocytes leading to nail thinning and brittleness, which may be related to this complex interaction.^6^ The total dystrophy observed in this case may represent the end spectrum of this neurotrophic process, in which chronic ischemia and altered neural signaling lead to irreversible matrix injury and loss of normal nail architecture.

Nail disorders secondary to neural injury, as CRPS, have scarce literature at present and may be underrecognized. Further studies of investigation evaluating nail disorders in subjects who suffered neural plexus traumas should be coordinated in order to understand better the physiopathology implicated in these processes.

## ORCID ID

Elisandra Bárbara Pontes Carlos: 0009-0001-4150-2598

Ivanka Miranda de Castro Martins: 0000-0002-1389-0749

Fábio Antônio de Andrade: 0009-0005-7876-9113

Laís Guimarães Souza: 0009-0001-4150-2598

## Financial support

CNPq (302789/2025-1) – Anna Carolina Miola is a research fellow at CNPq (PQ-C).

## Authors' contributions

Elisandra Bárbara Pontes Carlos: Conception and design of the study; data collection, analysis, and interpretation; article writing; critical review of the literature; final approval of the manuscript.

Ivanka Miranda de Castro Martins: Intellectual participation in the propaedeutic and/or therapeutic management of studied cases; critical review of the literature; final approval of the manuscript.

Fábio Antônio de Andrade: Intellectual participation in the propaedeutic and/or therapeutic management of studied cases; critical review of important intellectual content; critical review of the literature; final approval of the manuscript.

Laís Guimarães Souza: Critical review of important intellectual content; intellectual participation in the propaedeutic and/or therapeutic management of studied cases; critical review of the literature; final approval of the manuscript.

Anna Carolina Miola: Conception and design of the study; article writing; critical review of important intellectual content; effective participation in research guidance; intellectual participation in the propaedeutic and/or therapeutic management of studied cases; critical review of the literature; final approval of the final version of the manuscript.

## Research data availability

Does not apply.

## Conflicts of interest

None declared.
